# Ventricular Arrhythmia Originating from the Left Ventricular Papillary Muscles: Clinical Features and Technical Aspects

**DOI:** 10.19102/icrm.2018.090202

**Published:** 2018-02-15

**Authors:** Joseph A. Donnelly, Apoor Patel, Stuart J. Beldner

**Affiliations:** ^1^Department of Cardiology, Northwell Health System, Manhasset, NY, USA

**Keywords:** Ablation, cardiac imaging, papillary muscle, ventricular arrhythmia

## Abstract

The discovery, characterization, and ablation of the papillary muscles have evolved rapidly since the initial description in 2008. New innovations in pacemapping, intracardiac imaging, ablation catheters, and ablation methodologies have dramatically impacted the approach to the treatment of papillary muscle ventricular arrhythmias. This review provides an up-to-date summary of these methods, as well as guidance on how to integrate them into clinical practice.

## Introduction

The advent of electroanatomic mapping, intracardiac echocardiography, and multidetector computed tomography has led to the improvement of the identification and localization of endocavitary ventricular arrhythmias (VAs). Concomitantly, the role of VAs in the development of cardiomyopathies, and the potential reversal of cardiomyopathies, has expanded the indications for VA ablation. Endocavitary structures, dubbed the “fourth dimension,”^1^ had previously been neglected due to a lack of adequate techniques to accurately characterize them on available imaging technology. With imaging advances, the importance of these structures in the origin and propagation of VAs has been elucidated. Some centers report that up to 24% of VA ablations involve these endocavitary structures.^2^ In the right ventricle, the moderator band, tricuspid valve, papillary muscles, and false tendons have been identified as origins of VAs.^3,4^ In the left ventricle, the anterolateral and posterolateral papillary muscles have been confirmed as origins of ventricular tachycardia (VT). This review will focus on the arrhythmogenic nature of the left ventricular papillary muscles, their mechanisms, clinical identification, management, and future research.

Although previously postulated,^5–7^ the earliest reports of papillary VAs were in late 2007 to 2008.^8–10^ Since then, further descriptions, proposed mechanisms, diagnostic features, and treatment methodologies have evolved. Papillary VA is the second rarest form of VA originating from the left ventricle.^11^ The posterior papillary muscle is a more common location of origin of ventricular ectopy than the anterior papillary muscle. Papillary VAs have been described in both structurally normal and abnormal hearts.^8,9^ The papillary muscles demonstrate unique anatomy among structures of the heart. They are among the thickest endocardial structures, and fibers on the papillary muscles have separations, likely contributing to anisotropy.^12^

The original description of posterior papillary VA named seven key features, including (1) normal baseline electrocardiogram (ECG); (2) VA with a pattern of right bundle branch block (RBBB) with superior axis; (3) an inability to induce VA with programmed stimulation; (4) an inability to entrain; (5) inducibility with isoproterenol or epinephrine; (6) the earliest activation occurring in the papillary muscle; and (7) an absence of high-frequency potentials at the site of origin.^10^ Several other clinical features were later described. Papillary VAs were observed to rarely cause sustained VT,^13,14^ were often associated with exercise,^13^ and usually carried a benign prognosis. Later studies, however, found that premature ventricular contractions (PVCs) may be triggers of ventricular fibrillation^15^ and that papillary PVCs may induce a cardiomyopathy, which may be corrected with ablation.^16,17^

### Mechanism/etiology

These observations have led to a comprehensive understanding of the mechanism of papillary muscle VA. Papillary VA is an automatic or triggered focal arrhythmia, which often originates in the deep layers of the myocardium. This is supported by the pattern of induction by exercise, isoproterenol usage, lack of entrainment, tendency against sustained VT, late diastolic activation time at the ablation site, lack of fractionated potentials at the ablation site,^18^ acceleration with radiofrequency (RF) energy application,^13^ and the observation that the first beat of the tachycardia has the same morphology as subsequent beats. Fascicular VT and papillary VT remain closely linked, as the reentrant circuit of fascicular VT may involve the Purkinje fibers surrounding the papillary muscles.^19^

Papillary muscle VAs are often localized to deep myocardial origins. Evidence supporting a deep myocardial source include (1) the requirement for irrigated catheters for ablation; (2) poor results with pacemapping (completed far from the breakout site)^20^; and (3) a lack of interruption of VT with mechanical stimulation.^18^

Papillary VT can be seen in patients with both structurally normal and abnormal hearts. Some have noted the high prevalence of ischemic heart disease with low ejection fraction, suggesting that the left ventricular dilation and stretching of the papillary muscles may lead to papillary remodeling and ultimately, to myocardial automaticity.^21^ To further support this theory, there are reports of patients with separate VAs originating from both papillary muscles.^21^ Bogun et al. studied nine patients with a history of myocardial infarction with papillary VT and noted that magnetic resonance imaging (MRI) showed heterogeneous contrast uptake of the arrhythmogenic papillary muscles in some of these individuals.^9^ The same author group later studied patients without a history of myocardial infarction and found that 29% of these patients also had focal delayed enhancement in the culprit papillary muscles.^8^

### Surface electrocardiogram

Several studies to date have evaluated surface ECG characteristics to identify papillary muscle VA.^10,15,18,22^ VA originating from the papillary muscles must be differentiated from that from other sources of VA with RBBB morphology, such as fascicular VA or mitral annular VA.

Patients with papillary VA demonstrate an average QRS duration of 150 ms,^8^ and episodes of such are usually greater than 130 ms in duration. The RBBB pattern in V1 is not the typical rSR0 pattern of fascicular VT, but rather is often a monophasic R or qR pattern.^8^ In lead I and a VL, an R or Rs pattern may predominate.^23^ Positive concordance is not usually seen; however, the precordial transition occurs early.

**Figure 1:**
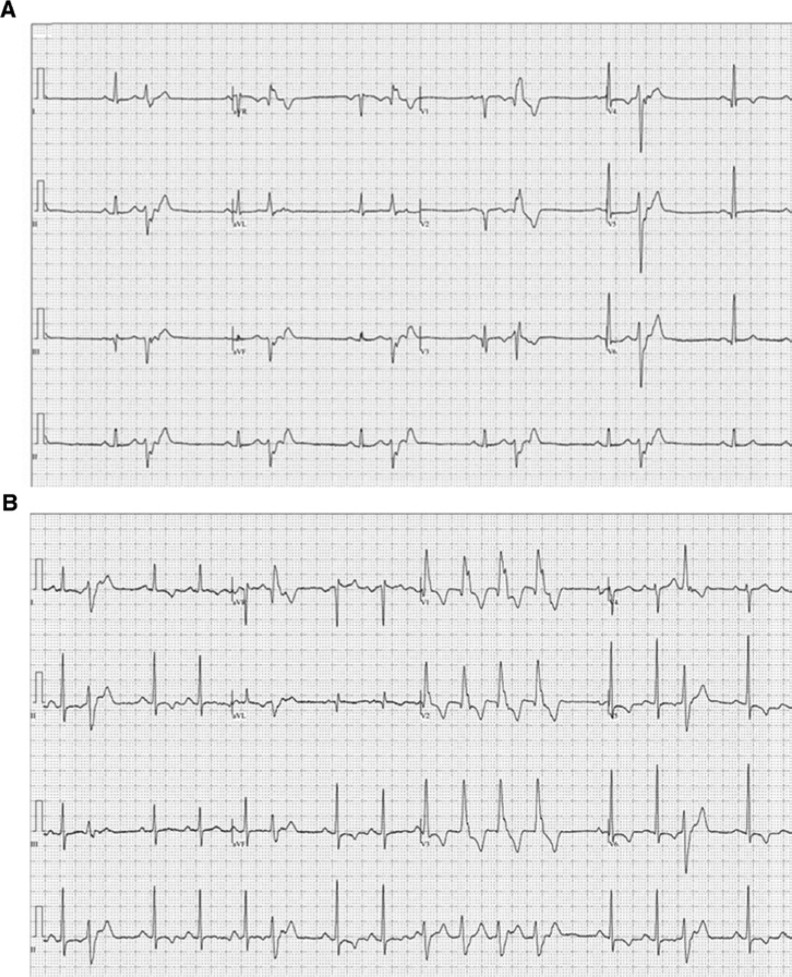
Surface ECGs of patients. **A:** Surface ECGs of patients with posteromedial papillary muscle VA. **B:** Surface ECGs of patients with anterolateral papillary muscle VA. These ECGs demonstrate the morphology and the variable presentation from isolated PVCs to the salvos of the VA. ECG: electrocardiogram; PVCs: premature ventricular contractions; VA: ventricular arrhythmia.

Papillary VA originating from the anterolateral papillary muscle has an RBBB morphology with an inferior or rightward axis. The precordial transition occurs between V3 and V5, often even before V1. There is also a qR or qr in lead aVR and an rS in V6 **(Figure 1A)**.

Papillary VT originating from the posteromedial papillary muscle has an RBBB morphology with a superior, leftward axis. R-wave notching is often present in more than three consecutive early precordial leads, possibly due to fractionated conduction through the papillary myocardium.^24^ The precordial transition also occurs in V3 to V5 and often before V1. The later precordial leads do not contain Q waves, and there is usually an rS in V6. There is never a qR or qRs in leads I, or a VL **(Figure 1B)**.^23^

While ventricular arrhythmias originating from the fascicles or mitral annulus may also have an RBBB pattern, with a similar axis to papillary VT, there are still significant differences. Features suggestive of fascicular VT include a QRS duration of less than 130 ms and an rsR′ pattern in V1.^25^ Fascicular VT is also associated with a qR or qRs pattern in I, or a VL. Posterior fascicular VT is associated with a small R wave in lead III. Also, in leads I, V5, and V6, there is an R/S ratio < 1.^19^ Anterior fascicular VT is associated with RS or QS in lead I, V5, and V6, with an R/S ratio < 1.^19^ Anterior mitral annular VTs are associated with positive precordial concordance due to the exit size near the base. Both anterior and posterior mitral VAs are associated with R ≥ S in V5.

Al’ Aref et al. devised a simple algorithm to differentiate these three VT origins using the surface ECG, based on the features of QRS duration, R/S ratio in V5, and precordial concordance and R/R0 ratio in V1, with an overall sensitivity and specificity of 83% to 100% for the various etiologies.^25^

## Papillary ventricular arrhythmias for the interventional electrophysiologist

### Anatomy of the endocavity

Given the unique anatomical aspects of endocavitary arrhythmias, the ablation of papillary VT is considered more technically challenging than that of other ventricular VAs.^8,10,13^ Anatomically, VA has been originating from all possible sites within the papillary muscles, the top near the chordae tendineae,^20^ the body, and the base. Successful ablation sites have been found on both the anterior and posterior sides of the papillary muscles.^18^ The posteromedial papillary muscles tend to accept successful ablation on the septal side, whereas the anterolateral papillary muscles have equal success with both anterior- and posterior-sided ablation.

Four major challenges specific to the ablation of papillary VT were first described by Doppalapudi et al.^10^ These include (1) the limited efficacy of pacemapping; (2) repeated changes in the morphology of arrhythmias during pacemapping; (3) difficulties in obtaining good contact between the catheter tip and papillary muscle; and (4) a frequent need for ablation on both sides of the papillary muscles in order to terminate the arrhythmia.

### Pacemapping

The mapping of papillary VA presents a distinct set of challenges. Given that the VA is caused by automatic or triggered activity, it is usually not sustained and cannot be entrained. In this regard, the operator must often rely on pacemapping. However, pacemapping is associated with inaccurate localization of the VA and failure to successfully terminate the arrhythmia.^18^ Yamada et al.^18^ demonstrated that despite excellent pacemapping scores of greater than 21, the RF energy delivered to the site of best match failed to terminate the PVC in 42% of patients. A change in PVC morphology was usually seen, however, likely due to the site of the origin of the VT being far removed from the breakout site.

Almost 50% of patients present with pleomorphic PVC morphologies.^8,18,26^ Yamada et al.^18^ reported that 50% of patients had multiple PVC morphologies. Possible mechanisms for this include (1) multiple origin sites or (2) multiple exit sites. The favored theory is that there are multiple exit sites, which is supported by the finding that the electrograms of VA demonstrate a fused form of multiple QRS morphologies. Also, in some patients, a single focus point demonstrated complete elimination upon ablation, supporting the theory of a single originating point.^18^

Chang et al. used pacemapping with an automatic matching algorithm and correlation score map to assist with multiple breakout sites. As with prior studies, they noted that 62% of patients had multiple breakout site morphologies. During the ablation of 13 patients, a total of 34 PVC morphologies were found. The authors reported a 100% long-term success rate.^27^

The weakness of pacemapping, which has been historically observed, is likely to be multifactorial. The two major limiting factors are technology and patient anatomy. Since 2008, pacemapping has improved from initially being visually guided, to currently employing computer-assisted matching algorithms. This is reflected in the increasing rates of ablation success seen over time, which are best demonstrated in the study by Chang et al.^27^ The second factor is the inherent anatomic limitations of pacemapping in the papillary muscle. The normal vigorous contractions of the papillary muscles limit catheter placement and stability, and their deep myocardial nature, as well as multiple exit sites, further limits the accuracy of pacemapping.

Electroanatomic mapping may also be attempted to ease the localization of VA origin; however, early diffuse activation sites may be distributed throughout the base of the papillary muscle. This may especially occur when the origination of the VA is in the mid-myocardium or epicardium.^21^

### Imaging

**Figure 2:**
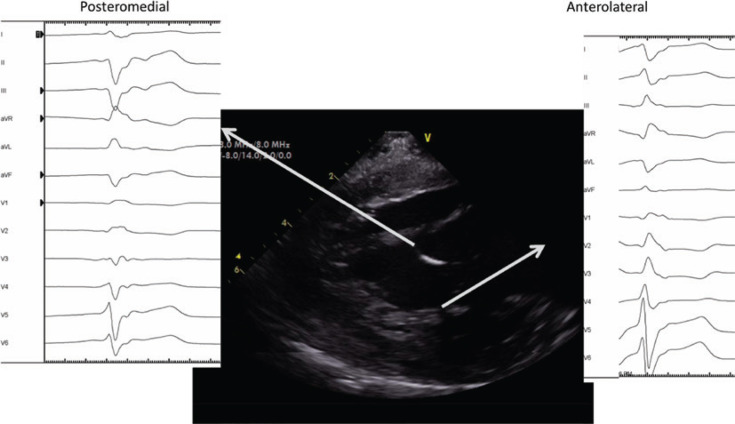
Intracardiac echocardiography anatomic correlation of posteromedial and anterolateral premature ventricular depolarizations.

**Figure 3:**
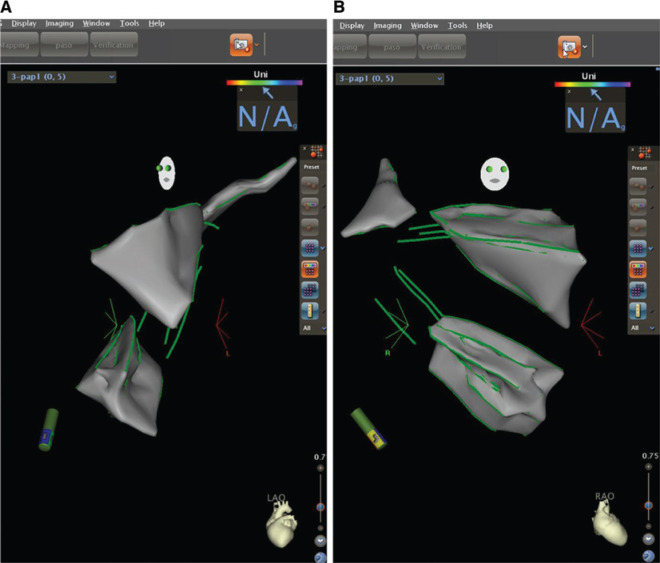
Three-dimensional echocardiograph reconstruction of the mitral valve and papillary muscles with CartoSOUND™ (Biosense Webster, Diamond Bar, CA, USA). **A:** Left anterior oblique view. **B:** Right anterior oblique view.

**Figure 4:**
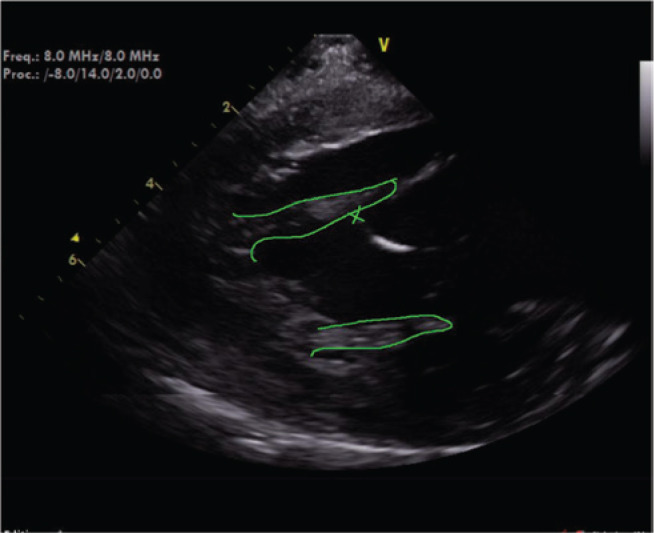
Intracardiac echocardiograph imaging demonstrating contact on the posteromedial papillary muscle.

Given the historic nature of intracardiac echocardiography (ICE) in the identification of papillary VA, its use has been paramount in the localization and confirmation of successful ablation sites. Proietti et al. described a case series of 16 patients undergoing RF ablation of the papillary muscles assisted by ICE imaging.^21^ In patients undergoing papillary VA ablation, the use of ICE guidance improved success rates from 28.5% to 62.5%. Our practice utilizes ICE with CartoSOUND™ (Biosense Webster, Diamond Bar, CA, USA) to create an anatomical map of the anterior and posterior papillary muscles **(Figure 2)**. Echo bright areas may also help to identify a site or location or origination. This may be consistent with the aforementioned areas of heterogeneous contrast uptake of the arrhythmogenic papillary muscles described by Bogun et al.^9^ We also use echocardiography to assess catheter contact with the desired papillary muscle **(Figures 3 and 4)**.

### Early potentials

Conflicting reports exist as to the presence and utility of early potentials seen during ablation. While some studies include the observation,^8^ others do not.^10^ Yamada et al.^11,13^ noted high-frequency potentials at successful ablation sites in patients during VT and identical appearing late potentials at the same site, respectively, in sinus rhythm. They suggested that the potentials may be used as markers of a successful ablation site. Their patients notably had a preponderance for sustained VT that was inconsistent with the findings of subsequent studies. Ban et al. later associated the finding with successful ablations, but also noted that low frequency potentials may represent far-field sensing of a deep myocardial source of VA.^24^ The conflicting reports of early potentials likely demonstrate the heterogenicity of depth of VA foci, with high frequency potentials being observed when the VA originate from more endocardial sites, versus in the case of deeper foci, in which early potentials may either be of a low frequency, or not present at all. Studies in patients with ischemic papillary VA have failed to report these high frequency potentials, perhaps due to the presence of scar tissue.^9^

### Role of cryoablation

RF application, most commonly via irrigated tipped catheters, has been the prevailing modality of papillary VA ablation, with the hypothesis of needing to create deep lesions in order to secure success. However, with the high recurrence rate, it has been postulated that the vigorous contractions associated with normally functioning papillary muscles make it difficult to maintain catheter contact during RF delivery. These contractions have remained problematic, despite the use of contact force measurement. An alternative approach using cryoablation has thus emerged, with various benefits.

Rivera et al. reported a small case series of successful cryoablations of papillary VAs in 2015.^28^ Ten patients underwent cryoablation for symptomatic, drug-refractory VAs using a transmitral approach. Cryoenergy was delivered to the earliest site of bipolar activity, earliest QS unipolar patterns, or earliest Purkinje network activity preceding the QRS. Electroanatomic mapping was performed with the EnSite™ NavX™ system (Abbott Laboratories, Chicago, IL, USA), and pacemapping was used. Immediate success rates were 100% non-inducibility and 100% catheter stability. One of the 10 patients had recurrence of VA on follow-up.

The same author group also published a retrospective series comparing 12 patients who underwent cryoablation with nine patients who underwent RF ablation. Antiarrhythmics were held in all patients post-ablation. With success defined as a ≥ 50% reduction in VAs, at a mean follow-up of 360 days, the cryoablation group had a 100% success rate versus a 56% success rate at 87 days follow-up in the RF group.^29^ Cryoablation was also associated with a decrease in multiple VA morphologies observed during ablation (0% versus 77%).

The success of cryoablation is likely due to improved catheter stability with cryoablation versus RF ablation, while the better catheter stability observed is likely to be due to an improved adherence of the catheter to the ablation site. There were fewer changes in VA morphology observed by Rivera et al., which may indicate the decreased thermal and mechanical effects that RF catheters might have on the myocardium.

The limitations of cryoablation remain problematic, including the tradeoff in catheter maneuverability, decreased lesion size and depth, and lack of integration with the CARTO™ system (Biosense Webster, Diamond Bar, CA, USA). However, it is compatible with the EnSite™ NavX™ system (Abbott Laboratories, Chicago, IL, USA). Marai et al. reported successful cryoablation of papillary VA using the combination of a cryocatheter with ICE alone, which is a feasible option in centers that only have the CARTO™ system (Biosense Webster, Diamond Bar, CA, USA) on hand.^30^

### Circumferential ablation

Wo et al. reported their experience with RF ablation using irrigated catheters with respect to the best pacemap site, augmented by circumferential ablation at the base of the papillary muscles. At a mean follow-up of 20 months, they had a 0% recurrence rate. Success was defined as the absence of VA on 24-hour Holter monitor three months after ablation, while the patient was off antiarrhythmic medications. No patients developed significant mitral regurgitation.^31^

## Risks

The risks of papillary muscle ablation are similar to those of other ventricular ablation procedures. The risk of stem pops may be worsened by restricted catheter mobility resulting from a tendency towards catheter wedging in the trabeculations between the myocardium and the papillary muscle insertion. This is particularly a concern with ablation performed at the base of the papillary muscles. Mitral regurgitation also remains a concern for the clinician; however, only two known case reports exist. One case involves a woman with preexisting mitral valve prolapse who worsened from moderate to severe after RF ablation.^32^ The other occurred in a 48-year-old man who developed severe mitral regurgitation requiring surgical correction.^33^

## Future research

Papillary muscle VT has been postulated to play a role in sudden cardiac arrest in patients with mitral valve prolapse.^34,35^ In a postmortem analysis of 43 young adults in whom mitral valve prolapse was the only abnormality on autopsy, all had RBBB pattern VAs on prior ECGs, and had papillary muscle fibrosis on histology. It is likely that there is a role of mechanical stretching of the papillary muscles involved in the genesis of papillary VA, as patients with mitral valve prolapse and VA from both papillary muscles have been described.^28^

## Our method/summary

We recommend an approach tailored to the patient’s anatomy. This includes localization of the VT using the surface ECG, as well as long-term ECG monitoring to assess for PVC burden, and for multiple PVC morphologies. The use of cardiac MRI may also confirm the loci of the VT, as 29% of papillary VTs demonstrated enhancement on MRI prior to electrophysiology study.^8^

The approach may be transseptal or retroaortic. No data exist on the efficacy differences between the two; we recommend a retroaortic approach for the anterolateral papillary muscle and a transseptal approach for the posteromedial papillary muscle. We find these approaches to be more suited anatomically to the respective papillary muscles, though success is possible using either approach.

Ablation should be assisted by imaging, which may include ICE, computed tomography imaging, or a combination of the two. Arrhythmia localization is performed via a combination of activation mapping and pacemapping. Pacemapping should be used with caution, however, due to the aforementioned studies. If an RF approach is to be used, we recommend using an irrigated catheter, targeting the exit site on the myocardium with the earliest bipolar activity preceding the QRS onset during arrhythmia, ideally more than 30 ms pre-QRS. If pacemapping is used, the operator should use the earliest activity obtained during the best example of pacemapping obtained. A target contact force of 10 g is recommended. If a papillary VT is confirmed, then irrigated catheters should be considered as they offer improved success rates due to the ability to ablate the deeper myocardial origin of the VT. However, irrigated catheters increase the risk of steam pops; thus, particular caution for rises in impedance should be undertaken. A recommended impedance drop is 10 ohms. Circumferential isolation of the papillary base may improve outcomes.

If poor results are achieved with RF ablation, or if there is significant catheter movement, then a cryoablation approach should be attempted. We recommend a transseptal approach with a cryocatheter. We also recommend assistance be provided via use of an available mapping system, or ICE if no compatible mapping system is available. We prefer the use of the EnSite™ NavX™ system (Abbott Laboratories, Chicago, IL, USA), though we have also used the CARTO™ system (Biosense Webster, Diamond Bar, CA, USA).

Features suggestive of a good target site for ablation include (1) early activation—more than 30 ms prior to QRS; (2) early sharp signals (the presence of which suggests endocardial location); (3) late potential in sinus rhythm that becomes pre-systolic during PVC/VT; (4) greater R-wave amplitude at distal bipolar ECG; (5) slow downstroke of initial Q wave on unipolar ECG; and (6) good catheter stability. In contrast, findings associated with ablation failure include (1) poor catheter stability; (2) a far-field appearance; and (3) reliance on pacemapping alone.

The discovery, characterization, and ablation of the papillary muscles have evolved rapidly since the initial description 10 years ago. Early studies had long-term success rates of ablation of 42% to 67% procedural success.^18,24^ With expanded use of cryoablation, circumferential ablation, and automatic pacemapping, current generation success rates are quoted to be as high as 90% to 100%.^27,29,31^ These advances have rapidly transitioned ablation of papillary VA from one of the least successful ablation procedures to one of high probability of cure. The lessons learned from papillary muscle arrhythmia are being applied to other endocavitary structures and may help to improve procedural success rates elsewhere in the future.
